# Intersection of Systematic Review Methodology with the NIH Reproducibility Initiative

**DOI:** 10.1289/ehp.1408671

**Published:** 2014-07-01

**Authors:** Kristina A. Thayer, Mary S. Wolfe, Andrew A. Rooney, Abee L. Boyles, John R. Bucher, Linda S. Birnbaum

**Affiliations:** 1 Division of the National Toxicology Program, and; 2 Office of the Director, National Institute of Environmental Health Sciences, National Institutes of Health, Department of Health and Human Services, Research Triangle Park, North Carolina, USA; E-mail: thayer@niehs.nih.gov

**Figure:**
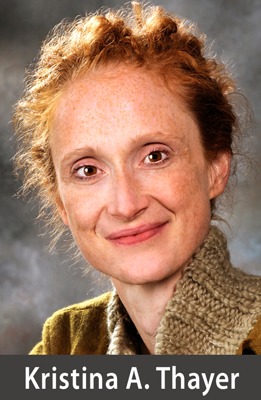
Kristina A. Thayer

**Figure:**
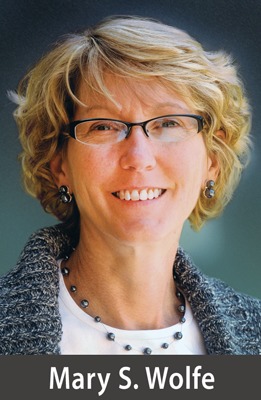
Mary S. Wolfe

**Figure:**
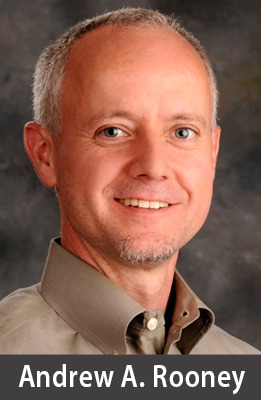
Andrew A. Rooney

**Figure:**
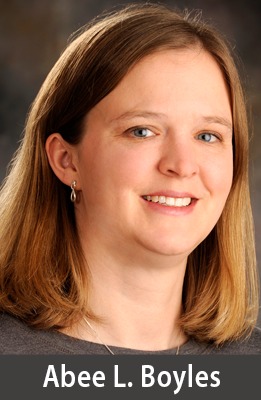
Abee L. Boyles

**Figure:**
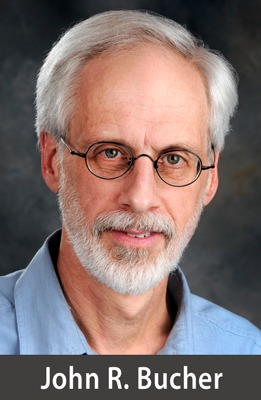
John R. Bucher

**Figure:**
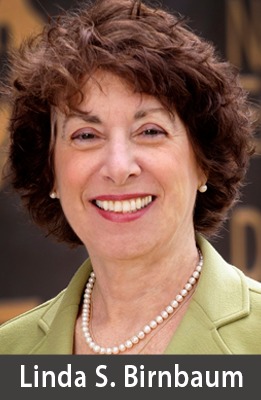
Linda S. Birnbaum

In a landmark 2005 paper published in *PLoS Medicine*, Ioannidis posited that “most current published research findings are false” (Ioannidis 2005). Consistent with this opinion are reports that drug development has been hindered and many clinical trials wasted based on published findings from preclinical studies that with further effort could not be reproduced (Begley and Ellis 2012). The National Institutes of Health (NIH) recently outlined a sweeping set of initiatives to address the lack of reproducibility of research findings (Collins and Tabak 2014). In this editorial we touch on current efforts to address the research reproducibility problem and propose that systematic review methodologies, which are being developed to assess confidence in the quality of evidence used in reaching public health decisions, could also be used to improve the reproducibly of research.

Reports and editorials in the biomedical literature have increasingly drawn attention to a disturbing lack of reproducibility of published scientific findings. Although poor reporting of key aspects of study methodology clearly contributes to the problem, other factors such as study conduct, may be equally or more important (Begley and Ellis 2012; Ioannidis 2005; Landis et al. 2012; Tsilidis et al. 2013). This situation has prompted actions by both the private and public sectors. These include the private Reproducibility Initiative collaboration between *Plos One* (http://www.plosone.org/), Science Exchange (https://www.scienceexchange.com/), figshare (http://figshare.com/), and Mendeley (http://www.mendeley.com/) (Nice 2013; Wadman 2013), which among other projects is attempting to replicate key findings from the 50 most impactful studies published in the field of cancer biology between 2010 and 2012. A major public effort is the NIH Initiative to Enhance Reproducibility and Transparency of Research Findings, which seeks to increase community awareness of the reproducibility problem, enhance formal training of investigators in elements of proper study design, improve the review of grant applications, and increase funding stability for investigators to enable them to use more appropriate and complex study designs (Tabak 2013). One planned activity of the NIH initiative is to develop a pilot training module on research integrity as it relates to experimental biases and study design. The intention is to provide specific guidance for researchers to improve the quality of their research publications by increasing their awareness of research practices that may affect the validity of their study findings. This guidance could also be used to improve both the grant proposal and journal peer-review stages to ensure more systematic and rigorous evaluation of both proposed and completed studies.

Hoojimans and Ritskes-Hoitinga (2013) recently published a progress report outlining a number of initiatives to address the reproducibility problem, specifically with respect to preclinical/experimental animal studies performed for translational research. Of course, experimental animal studies are critically important in many areas beyond drug development. Regulations to protect the public from harmful environmental exposures have historically relied heavily on the results of experimental animal studies. Within the larger area of environmental health sciences research, important evidence can also come from epidemiology studies of widely varying design, as well as from “mechanistic studies.” The consistent and transparent integration of this evidence to reach public health decisions is of immense international importance.

Implementing remedies to improve the reporting of key aspects of study methodology is perhaps the easiest challenge to address given that reporting quality checklists are available for clinical trials (Schulz et al. 2010), observational human studies (von Elm et al. 2008), animal studies (Hooijmans et al. 2010; Kilkenny et al. 2010), and *in vitro* studies (Schneider et al. 2009) (see also EQUATOR Network 2014). An increasing number of journals, including the *Nature* group, *Plos One*, and *Environmental Health Perspectives*, are now providing more explicit guidance to authors on items that should be reported when submitting papers.

A cornerstone of systematic review is the application of transparent, rigorous, objective, and reproducible methodology in a literature-based evaluation to identify, select, assess, and synthesize results of relevant studies. The application of systematic review methodology in an evaluation does not eliminate the need or the role for expert judgment. These methods do, however, offer a much-improved level of transparency for understanding the critical studies forming the basis for decisions and the overall confidence in the decision.

Establishing guidance to enable systematic assessment of the appropriateness of study design and conduct—or more generally, study quality—is challenging. Although there is a reasonable harmonization of approaches used to assess internal validity (risk of bias) for human clinical trials (Higgins and Green 2011), there is not currently a similar consensus on how to assess that the findings and conclusions drawn from observational human, experimental animal, and *in vitro* studies are a true reflection of the outcome of the study. For these types of data, ongoing methods development in the field of systematic review can help.

Interest has been growing in the fields of toxicology and pharmacology (National Research Council 2009; Rooney et al. 2014; Sena et al. 2007; Woodruff and Sutton 2011) in extending systematic review methods beyond the traditional area of human clinical trials to consider other evidence streams (observational human, experimental animal, and *in vitro* studies). For example, the National Toxicology Program (NTP) Office of Health Assessment and Translation (OHAT) has worked internationally to develop a formal approach for systematic review and evidence integration for literature-based evaluations through consultation with technical expert advisors, its scientific advisory committees, and with other agencies or programs that conduct literature-based assessments, as well as through public comment by stakeholders (Rooney et al. 2014). The Navigation Guide Work Group has developed a similar framework, and recent case studies support the feasibility of applying systematic review methods to environmental health evaluations. Because a key aspect in conducting a systematic review is to evaluate study quality, including internal validity or risk of bias for studies (Higgins and Green 2011), work by the NTP, Navigation Guide, and others is leading to the development of powerful risk-of-bias assessment tools applicable to a variety of human, animal, and mechanistic study designs. It is also leading to the development of methods to handle the assessment and integration of data within and across multiple evidence streams. Current systematic review methods under development differ in some respects but are substantively similar in approach. The flexible framework developed by OHAT (Rooney et al. 2014) allows evaluations to be specifically tailored to appropriately carry out environmental health assessments that include information derived from a diverse mix of study types and designs. This framework is envisioned to be continual, with refinements and improvements anticipated with use.

Investments in biomedical research today must result in improvements in quality of life in the future. Addressing the reproducibility of published scientific findings is of vital importance for maintaining the integrity of biomedical research. We believe that the widespread adoption and adherence to elements of systematic review throughout the entire scientific process, including study concept, grant writing and review, study performance, study reporting, and ultimately study utilization for reaching conclusions in environmental health sciences or any other area in biomedical research, can significantly improve both public health decisions and our return on scientific investment.
